# ILM flap coverage versus conventional ILM peeling for idiopathic full-thickness macular hole: a retrospective cohort study

**DOI:** 10.3389/fmed.2026.1827641

**Published:** 2026-08-07

**Authors:** Wendie Li, Yanyan Wang, Sangsang Wang, Jinghai Mao

**Affiliations:** Department of Ophthalmology, Ningbo Eye Hospital, Ningbo, Zhejiang, China

**Keywords:** ATO, ellipsoid zone, ILM peeling, macular hole, OCT, overlap weighting, temporal inverted internal limiting membrane flap, Type 1 closure

## Abstract

**Background:**

Idiopathic full-thickness macular hole (iFTMH) is treated by pars plana vitrectomy with internal limiting membrane (ILM) peeling. Conventional peeling achieves high closure in small holes but may yield inferior closure morphology in larger lesions. The temporal inverted ILM flap technique may improve closure quality, yet observational data stratified by Type 1 versus Type 2 closure require careful handling of baseline imbalance.

**Methods:**

We conducted a retrospective cohort study of 202 consecutive iFTMH eyes from 202 unique patients operated by a single team between January 2022 and December 2024. The strict primary-endpoint cohort comprised 198 eyes with 12-month OCT within a prespecified ± 4-week window (flap, *n* = 100; conventional peeling, *n* = 98). The primary endpoint was Type 1 closure at 12 months and the primary estimand was the average treatment effect in the overlap population (ATO). Propensity scores used pre-treatment covariates including hole dimensions, symptom duration, baseline BCVA, and calendar year. Common outcomes used g-computation with 1,000 bootstrap replicates (adjusted probabilities, risk differences, risk ratios); sparse-event outcomes used unweighted covariate-adjusted Firth-penalized regression as exploratory sensitivity analyses.

**Results:**

The flap group had larger baseline holes, worse baseline BCVA, and longer symptom duration (all *P* < 0.001). After overlap weighting, all standardized mean differences were <0.001, reflecting exact mean balance of included covariates (weighted effective sample size 111.5 of 202 eyes). Type 1 closure occurred in 82/100 (82.0%) flap versus 44/98 (44.9%) peeling eyes. In the ATO population the adjusted absolute risk difference was +33.5 percentage points (95% bootstrap CI +14.5 to +51.1), the adjusted risk ratio was 1.68 (95% CI 1.24–2.39), and the adjusted odds ratio was 4.84 (95% CI 1.95–12.00; *P* = 0.001). Exploratory secondary analyses suggested greater BCVA improvement, shorter EZ and ELM defect lengths, and fewer secondary surgeries in the flap group.

**Conclusion:**

In this retrospective single-center cohort with predominantly large iFTMH, temporal inverted ILM flap coverage was associated with a higher adjusted probability of Type 1 closure at 12 months than conventional ILM peeling within an overlap-population estimand. Exploratory secondary functional and microstructural findings should be interpreted as hypothesis-generating associations rather than confirmatory evidence.

## Introduction

Idiopathic full-thickness macular holes arise from abnormal vitreomacular traction that disrupts the neurosensory architecture of the fovea, producing central scotoma, metamorphopsia, and progressive visual impairment (1). Pars plana vitrectomy (PPV) with ILM peeling is the standard surgical treatment and achieves primary closure rates of 85%–98% in small holes up to approximately 400 μm in MLD (2–5). For larger holes (6–13), however, conventional ILM peeling is associated with a greater likelihood of Type 2 or flat-open closure morphology and incomplete restoration of the outer retinal layers (14).

The distinction between Type 1 and Type 2 closure is clinically meaningful. Type 1 closure indicates complete flat apposition of the foveal edges with no residual full-thickness defect, whereas Type 2 morphology indicates a flat-open or residual foveal defect configuration despite absence of a full-thickness open hole. Type 1 morphology is more closely linked to outer-retinal recovery, including ellipsoid zone (EZ) and external limiting membrane (ELM) restoration, than binary open/closed status alone (15).

The inverted ILM flap technique was originally described in 2010 for large macular holes; a temporal inverted ILM flap modification was subsequently reported in 2015, preserving a temporal pedicle while covering the foveal defect (16, 17). Because the present study used a temporal inverted flap rather than the classic circumferential inverted flap, we use ‘temporal inverted ILM flap coverage’ throughout. Although randomized and meta-analytic evidence supports the anatomical benefit of ILM flap techniques in larger holes (18–22), several clinically important questions remain underreported in routine surgical cohorts: whether closure quality rather than binary closure differs between approaches, whether clinically interpretable adjusted absolute effects can be estimated after marked baseline imbalance (23), and whether OCT-based outer-retinal microstructural outcomes (24) support the observed closure morphology.

We therefore compared temporal inverted ILM flap coverage with conventional ILM peeling in a consecutive iFTMH cohort using Type 1 closure at 12 months as the primary endpoint, applying overlap-weighted regression to address marked baseline imbalance and reporting adjusted absolute effects within an explicit overlap-population estimand.

## Materials and methods

### Study design and setting

This retrospective cohort study was conducted at a single tertiary vitreoretinal center and included consecutive iFTMH eyes operated by a single surgical team between January 2022 and December 2024. This was the actual recruitment/operation window for the analysed cohort, and no eyes operated outside this interval were included. The study adhered to the tenets of the Declaration of Helsinki and was approved by the Ethics Committee of Ningbo Eye Hospital (Approval No. 甬眼医伦审 2022 研第 015 号; Acceptance Number: 2022-49(K)-C2; Date of Approval: November 2, 2022). Written informed consent was obtained from all participants.

### Participants and unit of analysis

Inclusion criteria were: (i) OCT-confirmed idiopathic iFTMH, Gass stage 3 or 4; (ii) primary vitrectomy; and (iii) minimum 12-month postoperative follow-up. Exclusion criteria included secondary macular holes (axial myopia >6 dioptres (25), trauma, diabetic maculopathy), concurrent retinal detachment, previous ipsilateral vitreoretinal surgery, and silicone oil tamponade. No MLD cutoff was applied as an inclusion criterion; MLD was measured for all eyes and is also summarized according to OCT-based International Vitreomacular Traction Study Group (IVTS) size categories (1), with CLOSE-related morphology considered in the interpretation of large-hole closure patterns (26).

The patient-level denominator was rechecked before revision using patient-level identifiers rather than eye-level records alone. The full cohort comprised 202 eyes from 202 unique patients; all cases were unilateral study entries. No patient contributed both eyes, and no patient contributed one eye to each treatment group. Thus, the analytic unit was both the eye and the patient, with a one-to-one eye-patient correspondence. Within-patient clustering therefore did not apply, and the pre-specified one-eye-per-patient sensitivity analysis was, by construction, identical to the primary eye-level analysis.

### Study cohorts and analysis sets

Of 206 potentially eligible eyes, four did not pass the full-cohort eligibility review of preoperative and baseline data and were excluded during eligibility screening. The full cohort therefore comprised 202 eyes (temporal inverted ILM flap, *n* = 102; conventional peeling, *n* = 100) and was used for baseline description, safety outcomes, secondary vitreoretinal surgery, and sensitivity analyses. Within this full cohort, a further four eyes had their 12-month postoperative OCT assessment outside the prespecified ± 4-week window from the 12-month surgical anniversary and were excluded from the strict primary-endpoint cohort, yielding 198 eyes (temporal inverted ILM flap, *n* = 100; conventional peeling, *n* = 98). The last-available OCT sensitivity analysis included 201 eyes because one full-cohort eye did not have a classifiable last-available postoperative OCT image for closure-morphology assessment. This eye was retained for baseline, safety, and other ascertainable outcomes but could not contribute to OCT-based closure-morphology sensitivity analysis.

### Surgical technique and postoperative positioning

All operations were performed as 23-gauge pars plana vitrectomy under retrobulbar anesthesia. Following core vitrectomy and induction of posterior vitreous detachment when required, ILM staining was performed with 0.025% brilliant blue G for 60 s. In the temporal inverted ILM flap group, a single-layer temporal inverted ILM flap was fashioned by initiating peeling approximately 2.5–3 disk diameters temporal to the fovea, leaving a contiguous temporal pedicle attached at the hole margin, and folding the flap to cover the foveal defect. In the conventional peeling group, circumferential ILM removal extended approximately 2–2.5 disk diameters around the hole center and crossed the foveal region; a foveal-sparing or non-foveal-touch technique was not used in the control group during the study period.

All eyes received fluid-air exchange followed by gas tamponade with either 20% sulfur hexafluoride (SF6) or 14% perfluoropropane (C3F8) at the operating surgeon’s discretion. The choice of tamponade type was made intraoperatively after selection of the surgical approach and was based on hole size, surgical judgment, gas tolerance, and anticipated adherence to postoperative positioning. Combined phacoemulsification with intraocular lens implantation was performed in all phakic eyes in whom coexisting cataract was clinically significant. Because tamponade choice and combined phacoemulsification may function as co-interventions rather than baseline confounders, they were not included in the primary propensity model and were re-introduced only in a pre-specified sensitivity model.

Face-down positioning was recommended postoperatively according to the treating surgeon’s protocol and expected gas duration. Because exact adherence and duration were not uniformly captured in the retrospective records, positioning was not used as a model covariate and is discussed as a potential source of residual confounding (27).

### Outcome measures and OCT assessment

The primary endpoint was Type 1 closure at 12 months on the high-resolution horizontal foveal OCT B-scan. Type 1 closure was defined as complete flat apposition of the foveal edges without residual full-thickness foveal defect. Type 2 closure was defined as flat-open morphology with residual outer-retinal or foveal defect despite absence of a full-thickness open hole configuration. Flap-closure morphology in flap-treated eyes was recorded separately and classified as Type 1 or Type 2 according to the underlying foveal contour and residual defect pattern beneath the flap signal.

Key secondary endpoints were overall anatomical closure (any closed configuration), BCVA gain ≥2 lines on the logMAR chart, BCVA change from baseline to 12 months, absolute 12-month BCVA, EZ defect length, ELM defect length, secondary vitreoretinal surgery, and postoperative complications. These secondary outcomes were considered exploratory. EZ and ELM defect lengths were measured on the high-resolution horizontal foveal OCT B-scan (single-line scan through the foveal center, not a volume average) as the linear distance across the central discontinuity of the corresponding hyperreflective band. Each measurement was repeated three times by two masked retinal specialists who were not involved in the patients’ direct surgical or postoperative care. The mean of the three repeated measurements per reader was used; the mean of the two readers’ values was used as the final defect length for each eye. Disagreements in closure classification were adjudicated by a third masked specialist.

### Statistical analysis

Continuous variables are reported as mean ± SD and, for skewed variables (symptom duration, EZ and ELM defect lengths), additionally as median [IQR]. Categorical variables are reported as counts and percentages. Unadjusted comparisons used Welch’s *t*-test for approximately symmetric continuous variables with unequal variances and Wilcoxon rank-sum test for skewed continuous variables, and chi-square or Fisher’s exact test for categorical variables.

The primary estimand was the average treatment effect in the overlap population (ATO), representing the expected treatment contrast among eyes whose baseline characteristics made either surgical approach clinically plausible. Propensity scores were estimated using a logistic regression model including prespecified pre-treatment covariates: age, sex, lens status, MLD, BLD, hole height, symptom duration (28), baseline BCVA, and calendar year of surgery. Overlap weights were defined as *w* = 1 − e(X) for temporal inverted ILM flap eyes and *w* = e(X) for conventional peeling eyes, where e(X) is the estimated probability of receiving ILM flap coverage. Covariate balance was assessed using standardized mean differences (SMDs), propensity-score distributions, weight distributions, effective sample size (ESS), and common support. Exact mean balance of included propensity-model covariates after overlap weighting was expected under the fitted logistic propensity model and was distinguished from broader model adequacy or absence of residual confounding. Tamponade type and combined phacoemulsification were not included in the primary propensity model because they were selected intraoperatively after the surgical approach; they were examined only in a pre-specified sensitivity model.

Binary endpoints were analysed using overlap-weighted logistic regression. Because Type 1 closure and BCVA gain ≥2 lines are common outcomes, odds ratios were supplemented with adjusted marginal probabilities, adjusted absolute risk differences, and adjusted risk ratios estimated by g-computation with 1,000 bootstrap replicates and 95% percentile bootstrap confidence intervals. Sparse-event binary outcomes, including overall anatomical closure and secondary vitreoretinal surgery, were additionally evaluated using unweighted covariate-adjusted Firth-penalized logistic regression as exploratory sparse-data sensitivity analyses separate from the primary overlap-weighted ATO framework. These Firth models included treatment group and the same prespecified baseline covariates used in the propensity model and were used to reduce small-sample or separation bias in sparse binary endpoints. Continuous endpoints were analysed using overlap-weighted linear regression.

Pre-specified sensitivity analyses included: multivariable logistic regression without overlap weights, common-support restriction, last-available OCT in the full cohort, a calendar-period stratified analysis, a propensity model additionally including tamponade type and combined phacoemulsification, and a one-eye-per-patient sensitivity analysis (by construction identical to the primary analysis). Exploratory subgroup analyses were performed within prespecified strata (MLD < 500 vs. ≥500 μm, age ≤ 65 vs. >65 years, baseline BCVA ≤ vs. >median, symptom duration ≤ vs. >median, lens status, tamponade type). For each subgroup, a formal Wald-type interaction *P*-value was computed in an overlap-weighted logistic model including a treatment-by-covariate interaction term. Subgroup and sensitivity analyses were not adjusted for multiplicity and are considered exploratory.

Functional and microstructural outcome distributions are displayed descriptively in Figure 2. Primary inferential visual analyses focused on baseline-to-12-month BCVA change and absolute 12-month BCVA. Sample size was determined by the number of consecutive eligible cases during the study period; no formal prospective power calculation was performed because the study is retrospective. Precision is reported using 95% confidence intervals around the primary adjusted and marginal estimates. Baseline covariates were complete for all 202 eyes; postoperative BCVA was available for all 202 eyes at the 12-month visit; EZ and ELM defect lengths and closure morphology were measurable in all 198 strict-cohort eyes; secondary vitreoretinal surgery and postoperative complications were ascertained without loss to follow-up in the full cohort. Therefore complete-case analysis was used and no imputation was performed. Statistical significance was set at *P* < 0.05 (two-sided). Analyses were performed in R version 4.3.3.

**FIGURE 1:**
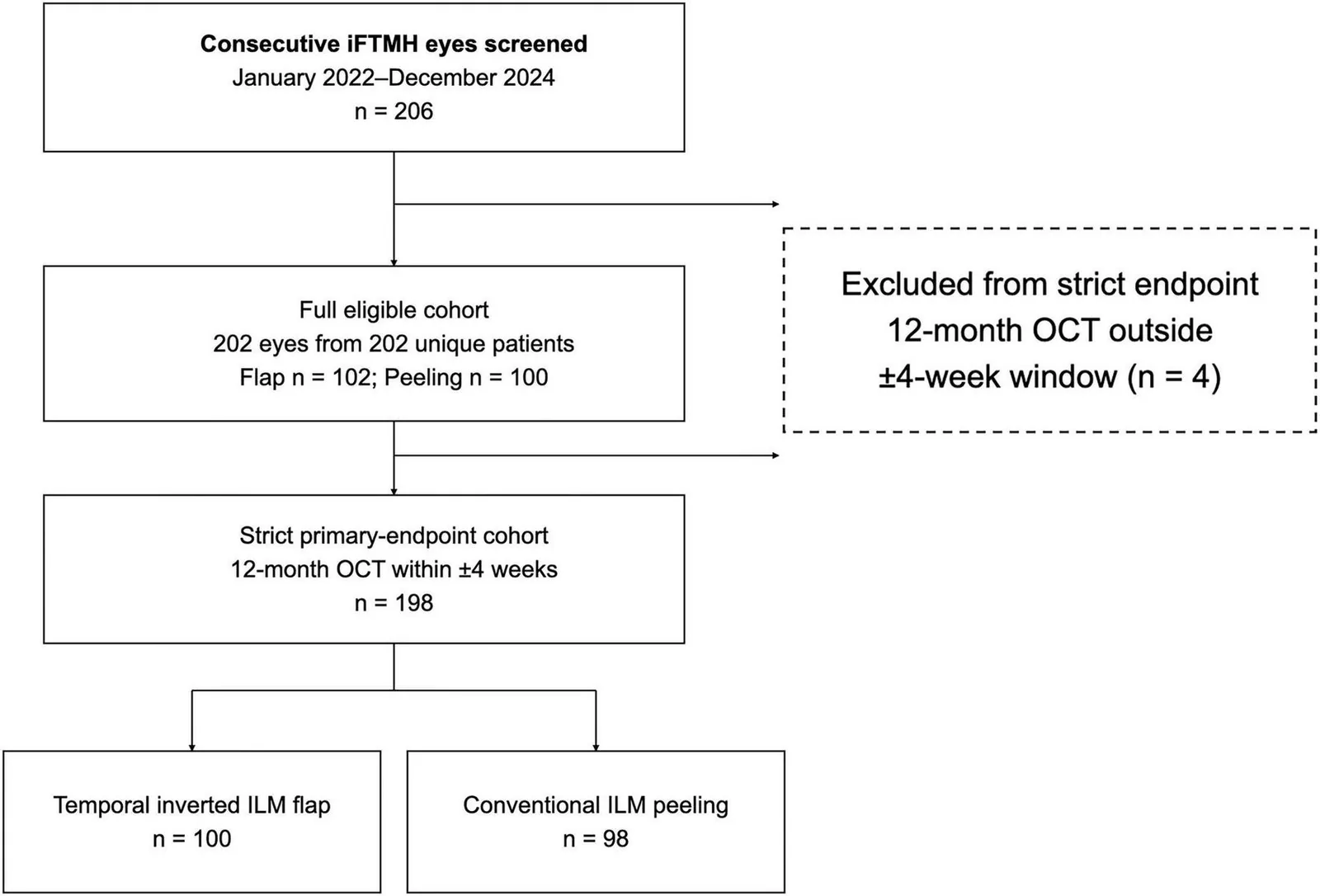
Study profile. Of 206 consecutive eyes with idiopathic full-thickness macular hole screened for eligibility, four eyes did not pass the full-cohort eligibility review of preoperative and baseline data and were excluded during screening, leaving a full cohort of 202 eyes from 202 unique patients (temporal inverted ILM flap, *n* = 102; conventional peeling, *n* = 100). A further four eyes were excluded from the strict primary-endpoint cohort because their 12-month postoperative OCT assessment fell outside the prespecified ± 4-week window from the 12-month surgical anniversary. The strict primary-endpoint cohort comprised 198 eyes (flap, *n* = 100; peeling, *n* = 98) and served as the basis for the primary and key secondary endpoint analyses.

**FIGURE 2:**
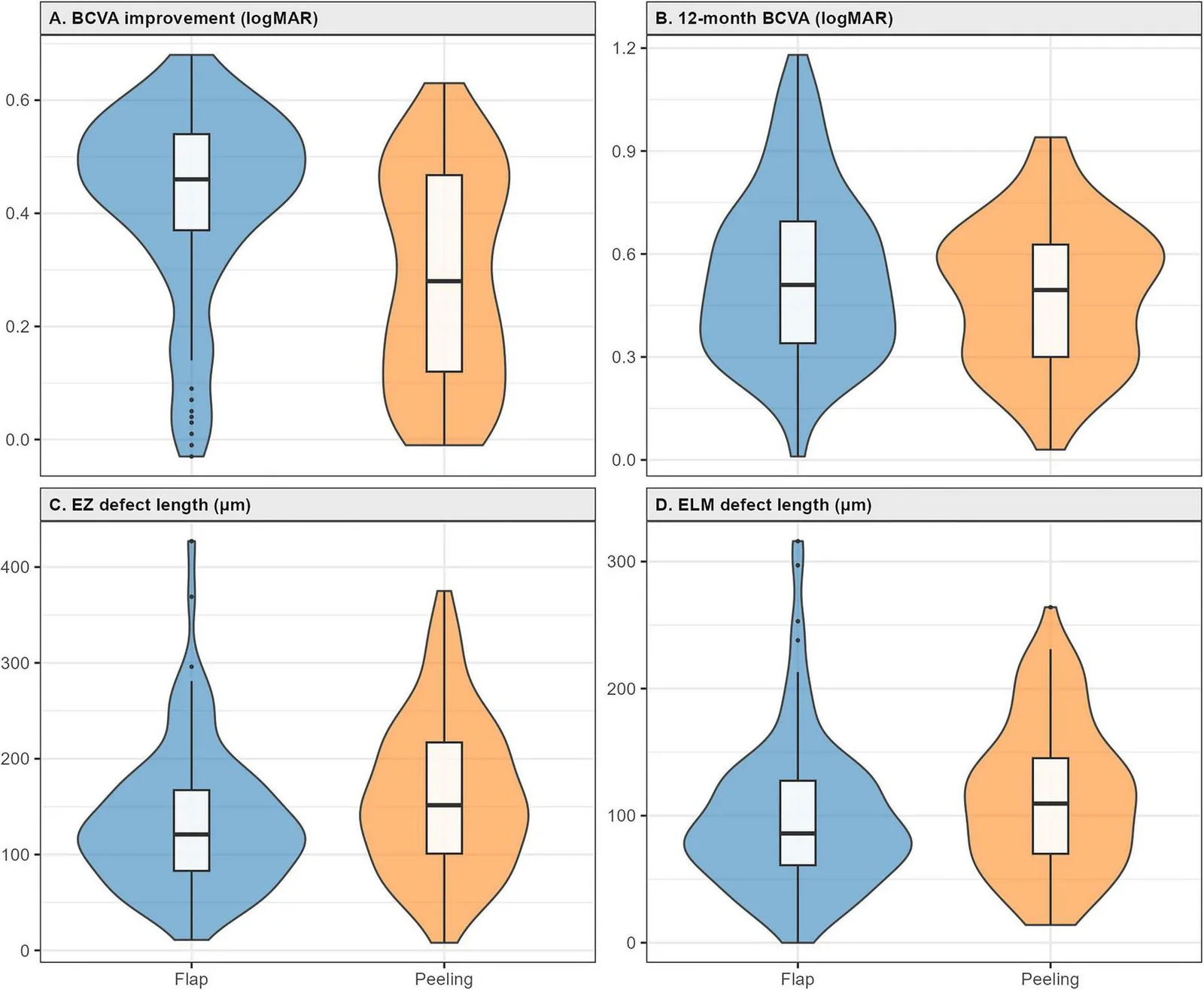
Distributions of functional and microstructural outcomes at 12 months: temporal inverted ILM flap coverage versus conventional ILM peeling. Violin plots (with embedded box plots showing the median and interquartile range) display, by group, the distribution of panel **(A)** BCVA improvement from baseline (logMAR reduction; higher values indicate greater gain), **(B)** absolute 12-month BCVA (logMAR; lower values indicate better acuity), **(C)** ellipsoid zone (EZ) defect length (micrometers), and **(D)** external limiting membrane (ELM) defect length (micrometers). The plotted distributions are descriptive; adjusted estimates are derived from overlap-weighted regression as described in the Methods. BCVA, best-corrected visual acuity; EZ, ellipsoid zone; ELM, external limiting membrane; ILM, internal limiting membrane.

## Results

### Study population and baseline characteristics

Of 206 screened eyes, 202 met full-cohort eligibility criteria and 198 met the strict 12-month endpoint window (Figure 1). The 202 eligible eyes corresponded to 202 unique patients, with one study eye per patient and no bilateral entries. Complete baseline covariate data were available for all 202 eyes. Four eyes had 12-month OCT assessments outside the prespecified endpoint window and were excluded from the strict primary-endpoint cohort but retained for full-cohort sensitivity and safety summaries.

Baseline characteristics are summarized in Table 1. The temporal inverted ILM flap group had larger and more complex holes and worse baseline vision than the conventional peeling group, reflecting real-world surgical case selection. Specifically, the flap group had larger MLD (625.8 ± 106.2 vs. 538.1 ± 83.8 μm), larger BLD (1298.1 ± 245.9 vs. 1102.0 ± 193.0 μm), greater hole height (317.2 ± 74.9 vs. 268.2 ± 61.7 μm), worse baseline BCVA (0.97 ± 0.15 vs. 0.77 ± 0.11 logMAR), and longer symptom duration (median 6 [IQR 4–8] vs. 3 [IQR 2–5] months; all *P* < 0.001). C3F8 tamponade was used more frequently in the flap group (77.5% vs. 43.0%; *P* < 0.001).

**TABLE 1:** Baseline characteristics of the full cohort (*n* = 202).

Characteristic	Temporal inverted ILM flap (*n* = 102)	Conventional peeling (*n* = 100)	*P*-value
Age, years	66.4 ± 5.9	64.9 ± 6.6	0.084
Female sex	70/102 (68.6%)	64/100 (64.0%)	0.552
Phakic lens status	66/102 (64.7%)	71/100 (71.0%)	0.368
Combined phacoemulsification	51/102 (50.0%)	45/100 (45.0%)	0.485
Symptom duration, months, median [IQR]	6 [4–8]	3 [2–5]	<0.001
Baseline BCVA, logMAR	0.97 ± 0.15	0.77 ± 0.11	<0.001
MLD, μm	625.8 ± 106.2	538.1 ± 83.8	<0.001
BLD, μm	1298.1 ± 245.9	1102.0 ± 193.0	<0.001
Hole height, μm	317.2 ± 74.9	268.2 ± 61.7	<0.001
IVTS large >400 μm	98/102 (96.1%)	88/100 (88.0%)	0.032
IVTS medium >250 to ≤400 μm	4/102 (3.9%)	12/100 (12.0%)	–
SF6 tamponade	23/102 (22.5%)	57/100 (57.0%)	<0.001
C3F8 tamponade	79/102 (77.5%)	43/100 (43.0%)	<0.001
Calendar year, median [IQR]	2023 [2022–2024]	2023 [2023–2024]	0.29

Data are mean ± SD, median [IQR], or n/N (%). *P*-values are from Welch’s *t*-test, Wilcoxon rank-sum test, chi-square test, or Fisher’s exact test, as appropriate. BCVA, best-corrected visual acuity; MLD, minimum linear diameter; BLD, base linear diameter; IVTS, International Vitreomacular Traction Study Group; SF6, sulfur hexafluoride; C3F8, perfluoropropane.

### Overlap-weighting diagnostics

After overlap weighting, all post-weighting absolute SMDs were <0.001, reflecting exact mean balance of the covariates included in the fitted logistic propensity-score model. This should be interpreted as balance of measured covariate means, not as proof of complete model adequacy or absence of residual confounding. The weighted effective sample size was 111.5 of 202 eyes, and the propensity-score distribution showed usable common support across the full covariate range. The common-support region (propensity scores 0.1–0.9) included 137 of 202 eyes in the full cohort and 134 of 198 in the strict primary-endpoint cohort. The exact weighting formula, maximum post-weighting SMD, ESS, and common-support details are reported in Table 2; per-covariate standardized mean differences before and after weighting are displayed as a Love plot, together with the propensity-score and overlap-weight distributions, in Supplementary Figure 1; and a compact covariate-balance and interaction summary is provided in Supplementary Tables 1, 2.

**TABLE 2:** Statistical diagnostics, missing-data summary, and OCT reliability.

Item	Result
Patient-level denominator	202 eyes from 202 unique patients; no bilateral study eyes
Actual recruitment/operation period	January 2022 to December 2024; no cases outside this interval were included
Primary estimand	Average treatment effect in the overlap population (ATO)
Overlap weights	*w* = 1 − e(X) for ILM flap eyes; *w* = e(X) for conventional peeling eyes, where e(X) is the estimated probability of receiving ILM flap coverage
Primary propensity covariates	Age, sex, lens status, MLD, BLD, hole height, symptom duration, baseline BCVA, calendar year of surgery
Maximum post-weighting absolute SMD	<0.001 (exact mean balance of included propensity-model covariates)
Weighted effective sample size	111.5 of 202 eyes
Common-support restriction (PS 0.1–0.9)	137 of 202 eyes (full cohort); 134 of 198 eyes (strict primary-endpoint cohort)
Missing baseline covariates	None in the full cohort (complete-case analysis)
12-month OCT outside ± 4-week window	4 of 202 eyes (excluded from strict primary-endpoint cohort)
Last-available OCT sensitivity analysis	201 of 202 eyes; one eye lacked a classifiable last-available postoperative OCT image for closure morphology
Postoperative BCVA at 12 months	Complete for all 202 eyes
EZ/ELM defect length at 12 months	Measurable in all 198 strict-cohort eyes
Secondary surgery / postoperative complications	Ascertained without loss to follow-up in the full cohort
Closure classification reliability (Cohen kappa)	0.82 (95% bootstrap CI 0.74–0.90); 15/198 required adjudication
EZ defect length reliability (ICC[2,1])	0.91 (95% CI 0.87–0.94)
ELM defect length reliability (ICC[2,1])	0.89 (95% CI 0.84–0.93)
Subgroup interaction *P*-values	MLD 0.86; baseline BCVA 0.43; symptom duration 0.25; lens status 0.21; tamponade 0.06 (all non-significant)

SMD, standardized mean difference; ESS, effective sample size; ICC, intraclass correlation coefficient; PS, propensity score; ATO, average treatment effect in the overlap population.

### Primary endpoint: Type 1 closure at 12 months

In the strict primary-endpoint cohort, Type 1 closure at 12 months occurred in 82 of 100 (82.0%) temporal inverted ILM flap eyes and 44 of 98 (44.9%) conventional peeling eyes (unadjusted *P* < 0.001). In the ATO population, the adjusted marginal probability of Type 1 closure was 82.7% (95% CI 70.5–93.4) in the flap group and 49.3% (95% CI 35.3–63.2) in the peeling group. The adjusted absolute risk difference was +33.5 percentage points (95% bootstrap CI +14.5 to +51.1), the adjusted risk ratio was 1.68 (95% CI 1.24–2.39), and the adjusted odds ratio was 4.84 (95% CI 1.95–12.00; *P* = 0.001). The adjusted odds ratio was stable across the pre-specified sensitivity analyses (Table 3). Flap-closure morphology was observed in 31 of 100 strict-cohort flap eyes in the early postoperative period and persisted at 12 months in 9 eyes; of these, 27 were classified as Type 1 and 4 as Type 2 closure according to the underlying foveal contour beneath the flap.

**TABLE 3:** Sensitivity analyses for Type 1 closure.

Analysis	Adjusted OR (95% CI)	Interpretation
Primary overlap-weighted model (ATO)	4.84 (1.95–12.00)	Reference
Multivariable logistic regression without overlap weights	4.86 (2.07–11.37)	Consistent
Common-support restriction (PS 0.1–0.9; *n* = 134)	6.74 (2.58–17.58)	Consistent
Last-available OCT in full cohort (*n* = 201)	4.25 (1.76–10.24)	Consistent
Added tamponade + phacoemulsification sensitivity model	5.43 (2.11–13.95)	Not attenuated
Calendar-period stratified analysis (2022–2023 vs. 2024)	5.03 (2.06–12.31)	Consistent
One eye per patient	4.84 (1.95–12.00)	Identical (no bilateral cases)

ORs compare temporal inverted ILM flap coverage with conventional ILM peeling. All sensitivity analyses are exploratory. The last-available OCT sensitivity analysis included 201 eyes because one full-cohort eye did not have a classifiable last-available postoperative OCT image for closure-morphology assessment; this eye was retained for non-OCT outcomes where ascertainable.

### Secondary anatomical, functional, and microstructural outcomes

Secondary outcomes are summarized in Table 4. Overall anatomical closure occurred in 97 of 100 (97.0%) flap eyes and 84 of 98 (85.7%) peeling eyes. Because of sparse events in the flap group, this exploratory outcome was analysed using unweighted covariate-adjusted Firth-penalized logistic regression (Firth OR 4.78, 95% CI 1.59–18.90). BCVA gain of at least 2 lines occurred in 86 of 100 (86.0%) flap eyes and 61 of 98 (62.2%) peeling eyes; the adjusted marginal probabilities were 88.3% versus 60.7%, with an adjusted absolute risk difference of +27.6 percentage points (95% CI +10.6 to +43.6). Mean BCVA improvement was greater in the flap group (0.43 ± 0.17 vs. 0.29 ± 0.19 logMAR reduction), and overlap-weighted linear regression yielded an adjusted beta of +0.18 logMAR (95% CI +0.11 to +0.25; *P* < 0.001). The adjusted beta for absolute 12-month BCVA was −0.18 logMAR (95% CI −0.25 to −0.12; *P* < 0.001).

**TABLE 4:** Primary and secondary outcomes at 12 months.

Outcome	Flap	Peeling	Adjusted estimate
Type 1 closure (primary)	82/100 (82.0%)	44/98 (44.9%)	Adjusted probabilities 82.7% vs. 49.3%; ARD +33.5 pp (95% bootstrap CI +14.5 to +51.1); RR 1.68 (1.24–2.39); OR 4.84 (1.95–12.00); *P* = 0.001
Overall anatomical closure (exploratory)	97/100 (97.0%)	84/98 (85.7%)	Unweighted covariate-adjusted Firth OR 4.78 (1.59–18.90); ARD +10.9 pp (95% CI +2.1 to +21.1)
BCVA gain ≥2 lines	86/100 (86.0%)	61/98 (62.2%)	Adjusted probabilities 88.3% vs. 60.7%; ARD +27.6 pp (95% CI +10.6 to +43.6); RR 1.46 (1.15–1.89); OR 4.68 (1.66–13.19); *P* = 0.004
BCVA improvement, logMAR	0.43 ± 0.17	0.29 ± 0.19	Adjusted beta +0.18 (95% CI +0.11 to +0.25); *P* < 0.001
12-month BCVA, logMAR	0.54 ± 0.25	0.48 ± 0.21	Adjusted beta −0.18 (95% CI −0.25 to −0.12); *P* < 0.001
EZ defect length, μm	133.9 ± 72.3	161.8 ± 79.7	Adjusted beta −43.44 (95% CI −68.62 to −18.26); *P* < 0.001
ELM defect length, μm	97.8 ± 57.2	115.3 ± 57.9	Adjusted beta −28.90 (95% CI −47.90 to −9.89); *P* < 0.001
Secondary vitreoretinal surgery (exploratory)	5/102 (4.9%)	20/100 (20.0%)	Unweighted covariate-adjusted Firth OR 0.22 (0.07–0.56); ARD −19.9 pp (95% CI −33.4 to −4.6)
Any postoperative complication (exploratory)	18/102 (17.6%)	25/100 (25.0%)	OR 0.54 (0.21–1.35); *P* = 0.19

Type 1 closure is the primary endpoint. Secondary outcomes are exploratory and were not adjusted for multiplicity. Firth models were unweighted covariate-adjusted sparse-event sensitivity analyses separate from the primary overlap-weighted ATO framework. Type 1 closure, overall anatomical closure, and BCVA outcomes are reported in the strict primary-endpoint cohort (flap *n* = 100; peeling *n* = 98); secondary vitreoretinal surgery and postoperative complications are reported in the full cohort (flap *n* = 102; peeling *n* = 100). For BCVA improvement, a positive beta indicates greater logMAR reduction (improvement). For 12-month BCVA, a negative beta indicates lower logMAR and better final acuity. For EZ/ELM defect lengths, a negative beta indicates a shorter defect. ARD, absolute risk difference; RR, risk ratio; OR, odds ratio; pp, percentage points; ATO, average treatment effect in the overlap population.

Outer-retinal microstructural outcomes also favored the flap group in exploratory analyses. Mean EZ defect length was 133.9 ± 72.3 μm in the flap group and 161.8 ± 79.7 μm in the peeling group; the adjusted beta was −43.44 μm (95% CI −68.62 to −18.26; *P* < 0.001). Mean ELM defect length was 97.8 ± 57.2 μm in the flap group and 115.3 ± 57.9 μm in the peeling group; the adjusted beta was −28.90 μm (95% CI −47.90 to −9.89; *P* < 0.001). These functional and microstructural outcomes were secondary, were not adjusted for multiplicity, and should be interpreted as exploratory.

Secondary vitreoretinal surgery was required in 5 of 102 (4.9%) flap eyes and 20 of 100 (20.0%) peeling eyes. The most common reasons were persistent or unclosed macular hole, recurrent macular hole after initial closure (29), and retinal detachment or retinal break (Table 5). This sparse-event endpoint was analysed using unweighted covariate-adjusted Firth-penalized logistic regression and is interpreted as exploratory. Postoperative complication categories are listed in Table 5. The composite ‘any postoperative complication’ rate was 17.6% in the flap group and 25.0% in the peeling group (OR 0.54, 95% CI 0.21–1.35; *P* = 0.19), with no significant difference after adjustment. No endophthalmitis occurred in either group.

**TABLE 5:** Reasons for secondary vitreoretinal surgery and postoperative complication categories (full cohort, *n* = 202).

Event category	Flap (*n* = 102)	Peeling (*n* = 100)	Comment
Persistent or unclosed macular hole requiring repeat vitrectomy/gas	3	15	Primary reason for repeat surgery
Recurrent macular hole after initial closure	1	3	Repeat vitrectomy or gas exchange
Retinal detachment or retinal break requiring vitreoretinal surgery	1	2	Surgery per standard retinal indication
Total secondary vitreoretinal surgery	5/102 (4.9%)	20/100 (20.0%)	Sparse-event exploratory endpoint
Cataract progression requiring surgery	6	9	Postoperative complication category
Transient intraocular pressure elevation	8	11	Controlled medically
Peripheral retinal tear treated with laser/cryotherapy	3	4	No progression to retinal detachment in either group
Epiretinal membrane / macular pucker noted during follow-up	1	1	Managed per clinical indication
Endophthalmitis	0	0	No case observed
Any postoperative complication	18/102 (17.6%)	25/100 (25.0%)	Full cohort safety summary

Interobserver reliability was good to excellent. Cohen’s kappa for three-level closure classification (Type 1/Type 2/open) was 0.82 (95% bootstrap CI 0.74–0.90), with 15 of 198 strict-cohort eyes requiring third-reader adjudication. ICC (1, 2) was 0.91 (95% CI 0.87–0.94) for EZ defect length and 0.89 (95% CI 0.84–0.93) for ELM defect length.

### Sensitivity and exploratory analyses

Sensitivity analyses were directionally consistent with the primary estimate (Table 3): multivariable logistic regression without overlap weights (OR 4.86, 95% CI 2.07–11.37), common-support restriction (OR 6.74, 95% CI 2.58–17.58), last-available OCT in the full cohort (*n* = 201; OR 4.25, 95% CI 1.76–10.24), calendar-period-stratified analysis (OR 5.03, 95% CI 2.06–12.31), a propensity model additionally including tamponade type and combined phacoemulsification (OR 5.43, 95% CI 2.11–13.95), and the one-eye-per-patient analysis (identical to the primary analysis because no patient contributed both eyes). The last-available OCT sensitivity analysis included 201 eyes because one full-cohort eye lacked a classifiable last-available postoperative OCT image for closure morphology; exclusion of this single eye did not change the direction of the estimate.

**FIGURE 3:**
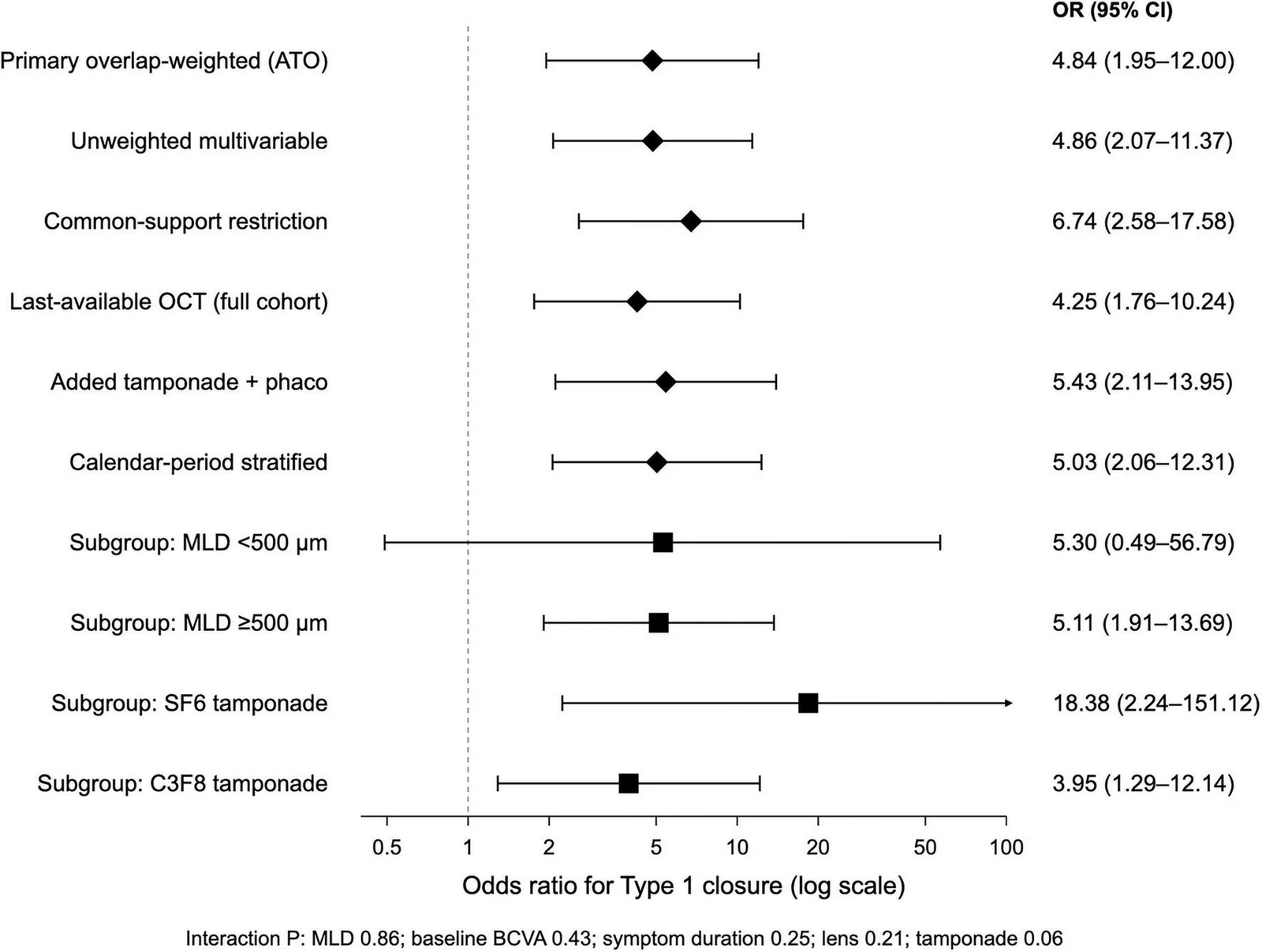
Sensitivity analyses and exploratory subgroup analyses for the primary endpoint (Type 1 closure). Odds ratios compare temporal inverted ILM flap coverage with conventional ILM peeling in each sensitivity-analysis scenario (upper panel) or in a pre-specified subgroup (lower panel). Subgroup analyses are exploratory; formal Wald-type interaction *P*-values from overlap-weighted logistic models including a treatment-by-covariate interaction term were uniformly non-significant (MLD P interaction = 0.86; baseline BCVA = 0.43; symptom duration = 0.25; lens status = 0.21; tamponade = 0.06). The MLD < 500 μm subgroup (*n* = 45) had an extremely wide CI (OR 5.30, 95% CI 0.49–56.79), and no subgroup-level clinical conclusion is drawn. Point size is proportional to the square root of the analytic sample. Diamond symbols indicate statistically significant results (*P* < 0.05); square symbols indicate non-significant results. OR, odds ratio; CI, confidence interval; MLD, minimum linear diameter; BCVA, best-corrected visual acuity; SF6, sulfur hexafluoride; C3F8, perfluoropropane.

Exploratory subgroup analyses are shown in Figure 3 and are treated as hypothesis-generating only. Formal Wald-type interaction tests in overlap-weighted logistic models that added a treatment-by-covariate interaction term were not significant for any pre-specified effect modifier: MLD (<500 vs. ≥500 μm) *P* interaction = 0.86; baseline BCVA (≤ vs. > median) *P* interaction = 0.43; symptom duration (≤ vs. >median) *P* interaction = 0.25; lens status (phakic vs. pseudophakic) *P* interaction = 0.21; tamponade (SF6 vs. C3F8) *P* interaction = 0.06. The MLD < 500 μm subgroup (*n* = 45) had an extremely wide confidence interval (OR 5.30, 95% CI 0.49–56.79), and no subgroup-level clinical conclusion is drawn.

## Discussion

In this retrospective single-center cohort of 202 unique patients with predominantly large iFTMH, temporal inverted ILM flap coverage was associated with a higher adjusted probability of Type 1 closure at 12 months than conventional ILM peeling, with consistent direction across pre-specified sensitivity analyses. The primary estimate is supported by adjusted marginal probabilities, an adjusted absolute risk difference of +33.5 percentage points, an adjusted risk ratio of 1.68, and an adjusted odds ratio of 4.84 within an explicitly defined overlap-population estimand. Secondary analyses suggested greater BCVA improvement and shorter EZ and ELM defect lengths in the flap group, but these secondary findings are exploratory and should not be interpreted as confirmatory evidence.

The revised analysis strengthens the clinical interpretability of the primary endpoint in several ways. First, Type 1 closure was retained as the primary endpoint because it captures closure quality rather than binary anatomical closure alone, which is more closely linked to outer-retinal recovery. Second, the unit of analysis was clarified at the patient level: after rechecking patient-level identifiers, all 202 eligible eyes corresponded to 202 unique patients, with no bilateral entries; consequently, within-patient clustering did not apply. Third, the ATO estimand was formally defined, allowing the adjusted effect to be interpreted within the clinically plausible overlap population rather than all eyes with iFTMH. Finally, the primary odds ratio was accompanied by adjusted marginal probabilities, an absolute risk difference, and a risk ratio, making the result more clinically interpretable.

The present study should not be interpreted as simply repeating the established finding that ILM flap techniques improve overall closure in large holes. Its added value is the combination of a strict 12-month Type 1 closure endpoint, OCT-based outer-retinal microstructural outcomes, masked OCT-reader reliability statistics, and clinically interpretable overlap-weighted effect estimates. The finding that secondary functional and microstructural outcomes trended in the same direction as the primary closure endpoint is biologically plausible, but these endpoints remain exploratory because of multiplicity, retrospective design, and residual confounding risk.

The large baseline imbalance between treatment groups reflects real-world surgical case selection: the flap group had larger holes, worse baseline BCVA, longer symptom duration, and more frequent C3F8 use. Overlap weighting was therefore central to the analysis. The exact post-weighting mean balance of included covariates supports the internal consistency of the propensity-score implementation, but it does not eliminate concerns about unmeasured confounding, functional-form misspecification, or evolving surgical practice over time.

Tamponade type and combined phacoemulsification warrant specific caution. Because they were selected intraoperatively after surgical approach selection, they may act as co-interventions or mediators rather than pure baseline confounders. For this reason, they were excluded from the primary propensity model and included only in a pre-specified sensitivity model. The sensitivity estimate was not attenuated, but this should not be interpreted as proof that tamponade choice had no clinical role.

The comparator procedure in this study was conventional circumferential ILM peeling that crossed the foveal region, rather than a foveal-sparing or non-foveal-touch technique (30). Therefore, the findings may not generalize to centers where foveal-sparing peeling, with preservation of central Muller cell cone integrity, is the standard comparator. This is acknowledged in the Limitations.

## Limitations

Several limitations require explicit acknowledgement. First, this is a retrospective non-randomized study from a single center; residual confounding from unmeasured variables (positioning adherence, surgeon learning-curve effects, detailed ILM-flap handling, and OCT acquisition variation) cannot be excluded. Although calendar year was included in the propensity model and a calendar-period sensitivity analysis produced results consistent with the primary estimate, temporal changes in surgical practice, case selection, imaging protocols, postoperative management, and surgeon learning-curve effects cannot be fully excluded in this retrospective surgical cohort. Second, the ATO estimand applies to eyes in the overlap population whose baseline covariates made either surgical approach clinically plausible and should not be generalized to all iFTMH eyes. Third, the moderate sample size contributes to uncertainty around some estimates, especially for sparse-event outcomes analysed with Firth-penalized regression. Fourth, secondary, sensitivity, and subgroup analyses were not adjusted for multiplicity and are reported as exploratory; subgroup interaction *P*-values were uniformly non-significant. Fifth, postoperative face-down positioning duration and adherence were not captured uniformly. Sixth, although representative serial OCT images can be clinically illustrative, the available ethics approval does not extend to publishing individual image panels; the revision therefore relies on explicit operational OCT definitions, masked-reader assessment, third-reader adjudication, kappa/ICC reliability statistics, and quantitative OCT endpoints rather than illustrative patient images. This limitation does not affect the primary outcome classification because closure morphology and EZ/ELM defect lengths were assessed on de-identified source OCT scans by masked readers before analysis.

## Conclusion

Temporal inverted ILM flap coverage was associated with a higher adjusted probability of Type 1 closure than conventional ILM peeling in a predominantly large-hole iFTMH cohort within an overlap-population estimand. BCVA, EZ/ELM defect length, secondary surgery, and complications were exploratory secondary outcomes and should be interpreted as hypothesis-generating associations rather than confirmatory evidence. The revised analysis clarifies the patient-level denominator, actual operation period, ATO estimand, overlap-weighting diagnostics, sparse-event modeling, OCT measurement reliability, and sensitivity analyses, while acknowledging that residual confounding and temporal practice effects cannot be fully excluded.

## Data Availability

The raw data supporting the conclusions of this article will be made available by the authors, without undue reservation.
