# Supplementary material for: ILM flap coverage versus conventional ILM peeling for idiopathic full-thickness macular hole: a retrospective cohort study
Source: Front Med (Lausanne). 2026 Aug 7;13:1827641. doi: 10.3389/fmed.2026.1827641 (PMC13493594; doi:10.3389/fmed.2026.1827641)
Supplement: Supplementary file 1 [file Table_1.docx]

**Supplementary Material**

# Supplementary Table S1. Exploratory subgroup interaction tests for Type 1 closure at 12 months

| **Pre-specified effect modifier** | **Categories** | **Interaction test model** | **P for interaction** | **Interpretation** |
| --- | --- | --- | --- | --- |
| Minimum linear diameter (MLD) | <500 vs. >=500 micrometres | Overlap-weighted logistic model with treatment x MLD category | 0.86 | No evidence of effect modification; subgroup result hypothesis-generating |
| Baseline BCVA | <= vs. > median | Overlap-weighted logistic model with treatment x baseline BCVA category | 0.43 | No evidence of effect modification |
| Symptom duration | <= vs. > median | Overlap-weighted logistic model with treatment x symptom-duration category | 0.25 | No evidence of effect modification |
| Lens status | Phakic vs. pseudophakic | Overlap-weighted logistic model with treatment x lens-status category | 0.21 | No evidence of effect modification |
| Tamponade type | SF6 vs. C3F8 | Overlap-weighted logistic model with treatment x tamponade type | 0.06 | No evidence of effect modification; interpreted cautiously because tamponade was a co-intervention |

# Supplementary Table S2. Submitted numeric diagnostics for overlap weighting

| **Diagnostic item** | **Submitted value / description** | **Purpose** |
| --- | --- | --- |
| Primary estimand | Average treatment effect in the overlap population (ATO) | Defines the target population as eyes for which either surgical approach was clinically plausible |
| Propensity-score model | Logistic model using age, sex, lens status, MLD, BLD, hole height, symptom duration, baseline BCVA, and calendar year of surgery | Restricts primary adjustment to pre-treatment covariates |
| Overlap-weight formula | w = 1 - e(X) for temporal inverted ILM flap eyes; w = e(X) for conventional peeling eyes | Targets the overlap population and down-weights treatment-deterministic observations |
| Maximum post-weighting absolute SMD | <0.001 (exact mean balance) across observed covariates | Indicates adequate observed covariate balance after weighting |
| Weighted effective sample size | 111.5 of 202 eyes | Evaluates information retained after weighting |
| Strict common support | 137 eyes (full cohort; 134 within the strict primary-endpoint cohort) with propensity scores between 0.1 and 0.9 | Sensitivity analysis assessing robustness within the strongest overlap zone |
| Missing baseline covariates | None among 202 full-cohort eyes | Supports complete-case analysis without imputation |
| Full-cohort eligibility screening | 4 of 206 screened eyes excluded for not passing the preoperative/baseline eligibility review | Explains the full cohort of 202 eyes (206 screened minus 4) |
| Strict-window OCT unavailable | 4 eyes outside the prespecified +/-4-week 12-month OCT window | Explains the strict primary-endpoint cohort of 198 eyes |

# Supplementary Note S1. Rationale for not adding patient-level OCT image panels in this submission

The reviewers requested representative OCT images to illustrate progression from preoperative macular hole morphology to postoperative Type 1 and Type 2 closure. The authors agree that such images are clinically helpful. However, patient-level OCT image export for publication was not available under the authors' current de-identification workflow for this submission, and the original submission statement specified that no potentially identifiable images or data were presented. To avoid introducing inadequately de-identified patient images, the revision does not rely on illustrative patient images. Instead, the manuscript provides explicit operational OCT definitions, masked-reader assessment, repeated measurements, adjudication procedures, Cohen's kappa for closure classification, ICCs for EZ and ELM measurements, and quantitative OCT endpoints. This limitation is acknowledged in the Discussion.

This note does not affect the primary endpoint classification: Type 1 and Type 2 closure were determined from source OCT scans by masked retinal specialists before analysis, and EZ/ELM defect lengths were measured quantitatively on the high-resolution horizontal foveal B-scan.

Supplementary Figure S1. Overlap-weighting diagnostics for the primary 9-covariate ATO model.

(A) Love plot of per-covariate absolute standardised mean differences before (open) and after (filled) overlap weighting; after weighting all values are <0.001 (exact mean balance). (B) Propensity-score distribution by treatment group. (C) Overlap-weight distribution by group. Weighted effective sample size 111.5 of 202 eyes; common-support region 137 of 202 eyes.
